# Comparing complementary alternative treatment for chronic shoulder pain of myofascial origin: Collateral meridian therapy versus local tender area-related meridians therapy

**DOI:** 10.1097/MD.0000000000004634

**Published:** 2016-09-02

**Authors:** Ru-Yu Pan, Yung-Chi Hsu, Chih-Shung Wong, Shinn-Long Lin, Tsung-Ying Li, Chen-Hwan Cherng, Shan-Chi Ko, Chun-Chang Yeh

**Affiliations:** a Department of Anesthesiology and Integrated Pain Management Center; b Department of Orthopedics, Tri-Service General Hospital and National Defense Medical Center; c Department of Anesthesiology, Cathay General Hospital; d Department of Physical Medicine and Rehabilitation, Tri-Service General Hospital and National Defense Medical Center, Taipei, Taiwan, Republic of China; e Ginza Hospital, Tokyo, Japan.

**Keywords:** collateral meridian therapy, shoulder pain and disability index (SPADI), visual analogue scale (VAS)

## Abstract

The aim of this study was to compare the short-term outcomes between 2 different treatments for unilateral chronic shoulder pain of myofascial origin, that is, local tender area related meridians (LTARMs) treatment and collateral meridian therapy (CMT), which were performed 6 times over a period of 4 weeks.

Seventy patients with unilateral shoulder pain of chronic myofascial origin were enrolled. The patients were randomly assigned to 2 different treatment groups: 1 group received CMT (n = 35) and the other received LTARM (n = 35). Before and after the 2 treatment processes, all patients rated their overall pain intensity on a visual analogue scale (VAS) and a validated 13-question shoulder pain and disability index (SPADI) questionnaire was used to measure shoulder pain and functional impairment after therapy for 4 weeks.

After CMT, the pain intensity was reduced after CMT. VAS score is reduced from 5.90 ± 2.07 (a mean of 5.90 and standard deviation of 2.07) to 3.39 ± 1.2. This was verified by the SPADI pain subscale scores (from 0.58 ± 0.193 to 0.33 ± 0.14). The pain-relief effect of CMT was significantly better than that of LTARM (VAS score from 5.78 ± 1.64 to 4.58 ± 1.40; *P* < 0.005; SPADI pain subscale score from 0.58 ± 0.16 to 0.45 ± 0.14, *P* < 0.001). In addition, the VAS scores of patients changed considerably in the CMT group after 4 weeks of treatment, where 63% of patients felt no or mild pain, whereas the VAS scores for moderate pain were even higher in the LTARM group in 75% of patients (*P* < 0.001). Moreover, the SPADI disability subscale scores improved significantly in the CMT group because of their greater mobility associated with shoulder impairment (disability score: from 0.58 ± 0.20 to 0.35 ± 0.14) than those in the LTARM group (disability score: from 0.55 ± 0.17 to 0.44 ± 0.14, *P* < 0.001).

CMT may be more effective in reducing chronic shoulder pain of myofascial origin than the LTARM treatment, where treatment with the former resulted in better functional recovery after 4 weeks than the latter.

## 1 Introduction

Shoulder pain is a considerable burden on patients and their families,^[1]^ and it is the third most common cause of musculoskeletal complaints in clinical care.^[2]^ There are no gold standard criteria for shoulder pain diagnosis, and thus significant differences in prevalence (from 6.9% to 26%) have been noted.^[3]^ Various treatments for shoulder pain of myofascial origin have been reviewed, including dry needle,^[4]^ acupuncture,^[5]^ multimodal treatment,^[6]^ low-level laser therapy,^[7]^ and fascial manipulation.^[8]^ In general, these are local treatments that focus on the affected region and they are only moderately effective in improving the function of the painful shoulder and reducing pain.^[9]^ Collateral meridian therapy (CMT) is a recently developed technique derived from traditional Chinese medicine, but it differs considerably from traditional Chinese acupuncture.^[10]^ In particular, CMT is a 2-point manipulation technique that involves operations on the control point (C-point) and the functional point (F-point) located in relevant healthy meridians, which are distal to the associated disease-related meridians. Thus, CMT redirects, removes, reduces, or strengthens the body energy flows (“Qi”) to achieve pain relief and to restore homogeneity.^[10]^

An important characteristic of CMT is that it is usually applied to distal acupoints or meridians located away from the lesion site, and clinically, CMT appears to be more effective than direct local acupressure or treatment of the affected meridian alone. Previously, we have successfully applied CMT to treat musculoskeletal pain, such as postregional anesthesia/analgesia backache and postlaparoscopic shoulder-tip pain.^[11,12]^ In the latter study, we showed that CMT was even more effective than acupressure on the local tender area.^[12]^

In the present pilot study, we compared 2 treatments for unilateral chronic shoulder pain of myofascial origin, that is, local tender area related meridians (LTARMs) treatment and CMT for a period of 4 weeks. We performed evaluations to determine which was the most effective for shoulder pain relief and functional recovery.

## 2 Methods

### 2.1 Patients

We received approval from Tri-Service General Hospital institutional review board (TSGHIRB No: 98-04-002) and performed in compliance with the Helsinki Declaration. The patients were outpatients at pain clinics and rehabilitation division in the Tri-Service General Hospital, Taiwan. All patients were examined according to a standardized assessment process to ensure that their pain was musculoskeletal in origin. After careful evaluation, including physical examinations, X-ray, and sonography of shoulder joints, patients aged between 20 and 65 years who had complained of unilateral shoulder pains for at least 3 months were included in the study. All participants were asked not to take pain-killers for at least 1 week before the study, as well as during the study period. Those who refused or failed to do so were excluded. Over the course of the trial, investigator-supplied paracetamol was the only pain-killer allowed for additional pain relief. Patients with the following conditions were also excluded from our study: cervical neuropathy/surgery or peripheral neuropathy; bone fractures; unstable joints/impingement syndrome; contraindications that excluded acupuncture or electrical stimulation, such as pregnancy and cardiac pacemakers; partial or full-thickness tear of rotator cuff; significant impairment of sensation; or fibromyalgia.

Before the enrollment, the patients were informed that there were 2 commonly applied meridian therapies, viz. CMT and LTARM, and that both were effective methods in relieving chronic pain. But there was no hint whatsoever as to which might be a better treatment than the other. The patients were informed that they would undergo meridian therapies for chronic shoulder pain, but they had no idea whether they received CMT or LTARM, as both procedures involved using an electrotherapy stimulator, attaching electrodes to certain areas, and/or acupoints of the patients. For the participants, there was no distinguishing between the 2 therapeutic processes. Using a computer-based randomization program, patients were allocated at random to either the CMT or LTARM groups. All participants were asked not to reveal their therapeutic details to the research assistant, and they were not aware that they would be which one therapeutic group. An independent research assistant who was engrossed in the study and remained “blind” to group allocation executed outcome assessments. The patients, research assistant, and the statistical analysts who reviewed the outcome were all blinded to the group assignments.

### 2.2 Complementary alternative treatment techniques

**Figure 1:**
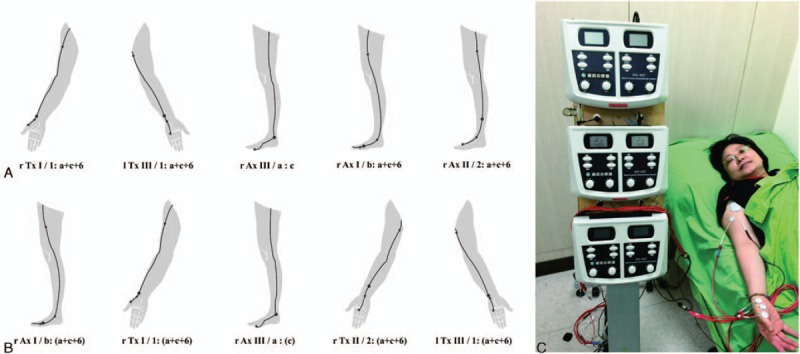
Treatment procedures for chronic left shoulder pain of myofascial origin. (A, B) Maneuver A (reduction method) and maneuver B (enhancement method), respectively. (C) shows a patient under CMT treatment using EES. For chronic right shoulder pain, the treatment procedures were performed in the opposite manner.

**Figure 2:**
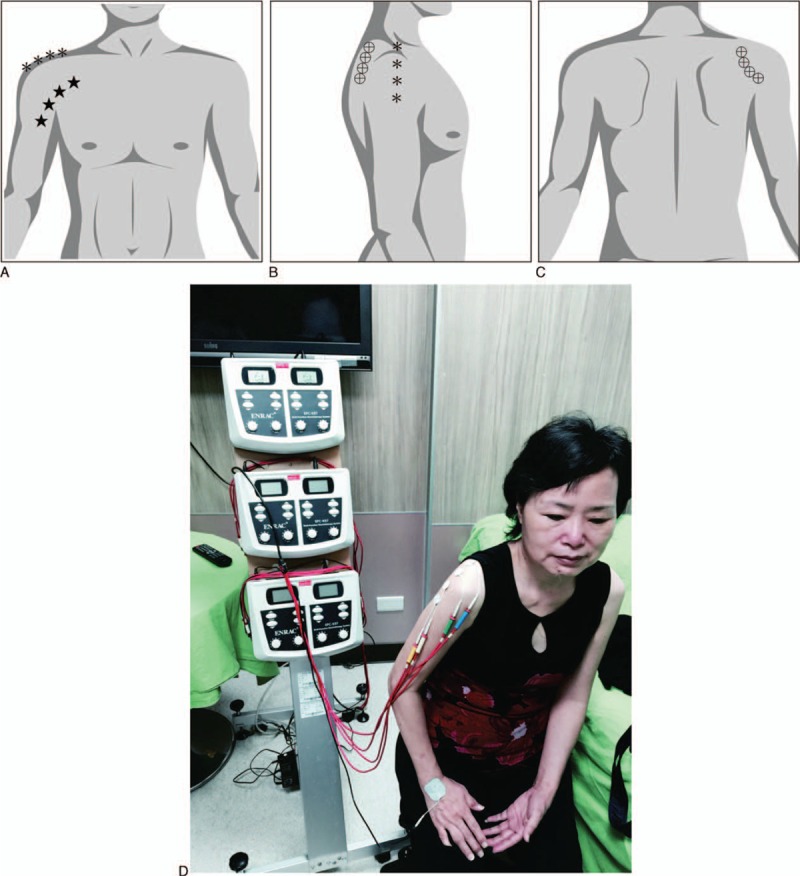
Treatment procedures in the LTARM group using EES, applied on local tender areas, covering anterior, superior-lateral, and posterior shoulder parts, respectively (A–C). Treatment for each part lasted 20 minutes, totaling about 60 minutes. (D) shows a patient under LPARM treatment using EES.

All of the treatment procedures for the 2 groups were prescribed by the same pain specialist well-trained in CMT advanced course. All subjects were treated in a supine or sitting position. In the CMT group, the specialist diagnosed the disease meridians in the affected shoulder and wrote 2-step prescriptions. The treatment procedures were conducted by a well-trained nurse, who was prohibited not to reveal any therapeutic information to the participants, using an ENRAC electrotherapy stimulator (EES; “GEMORE”, Multi-Function Electrotherapy Stimulator; GEMORE Co Ltd, New Taipei City, Taiwan) for about 60 minutes in a 2-step maneuver, that is, maneuver A (reduction method, Table 1 and Fig. 1A) followed by maneuver B (enhancement method, Table 1, Fig. 1B, C). The LTARM group also underwent treatment procedures using EES for about 60 minutes, but it was applied only to local tender areas, covering anterior, superior-lateral, and posterior shoulder parts, respectively, for 20 minutes time each (Fig. 2A∼D). The electrodes were firmly attached to the skin with adhesive. The intensity of electrical stimulation during treatments was determined by patients as the level at which each patient felt strong, but not painful, paresthesia in either distal acupoints in the CMT group, or local tender points in the LTARM group.

**Table 1:**
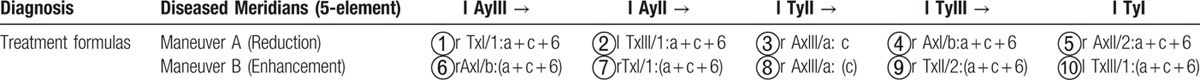
An example for the diagnosis of affected diseased meridians and the corresponding 2-step treatment formulae for CMT group patients with chronic left shoulder pain.

### 2.3 Endpoints

Before and after 4 weeks of therapy (6 times), all of the patients were asked to rate their pain on a visual analogue scale (VAS) and a questionnaire was also completed. The VAS score assessed the overall pain intensity,^[13]^ and the validated 13-question shoulder pain and disability index (SPADI) questionnaire was completed to measure shoulder pain and functional impairment by a research assistant, who was blinded to the exact treatment procedures.^[14,15]^

### 2.4 Statistical analysis

The statistical analyst was blinded to both the subjects and the investigators. The statistical analyses were performed using the Statistical Package for the Social Sciences (SPSS 20.0 for Windows; IBM Corp., Armonk, NY). Independent *t* tests were used to compare differences in age, height, weight, and duration of pain between patients who received CMT therapy and LTARM therapy. We performed Chi-square tests to explore differences in variables related to sex between patients who received CMT therapy and LTARM therapy. In addition, independent *t* tests were conducted to compare differences in the overall VAS scores between patients who received CMT therapy and LTARM therapy. Furthermore, a Chi-square test was used to compare differences in pain levels between patients who received CMT therapy and LTARM therapy at baseline and after 4 weeks. Finally, we used independent *t* tests to compare the differences in SPADI ratings between patients who received CMT therapy and LTARM therapy at baseline and after 4 weeks. Differences were considered significant for 2-sided *P* values <0.05.

## 3 Results

**Figure 3:**
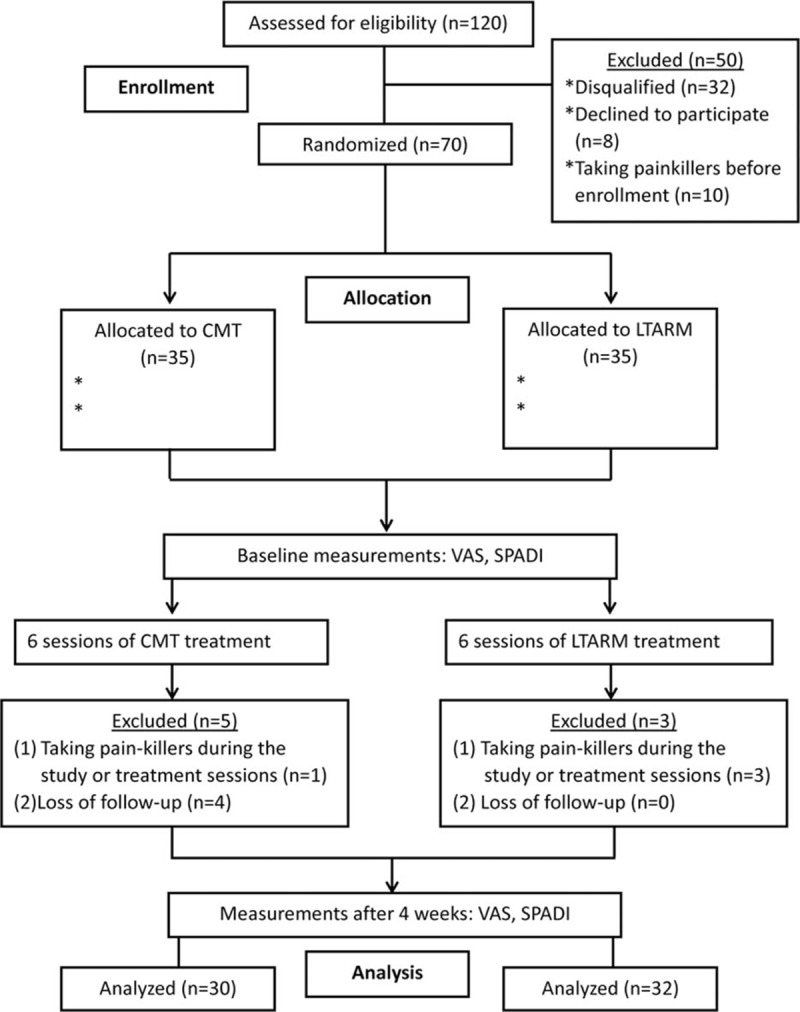
Flowchart outlining the patient eligibility, randomization, and analysis processes.

In total, we assessed 120 patients for eligibility and 70 patients were enrolled to the study. These patients were randomly assigned to the CMT and LTARM groups, with 35 patients in each group. Subsequently, 8 of these 70 patients withdrew from the study, and thus, 62 patients qualified for the study in the end. Figure 3 shows the number of patients at each stage and the reasons for exclusion from our study. The patient characteristics are detailed in Table 2, which show that there were no obvious differences in the baseline values before treatments. After 6 treatments, 5 patients were excluded from the CMT group, that is, 1 took pain-killers during the study and 4 were lost at follow-up. In the LTARM group, 3 were excluded because they took pain-killers during the treatments. The overall changes in the pain levels according to the VAS scores after CMT and LTARM are presented in Table 3. Before the treatments, there was no statistical difference in the VAS scores at baseline between the 2 groups. However, after the treatments, the VAS scores for the CMT and LTARM groups differed significantly, that is, 3.39 ± 1.41 (a mean of 3.39 and standard deviation of 1.41) and 4.58 ± 1.40, respectively. Moreover, the VAS scores for the CMT group decreased by 2.51 ± 1.99 and those for the LTARM group decreased by 1.21 ± 1.47, thereby demonstrating that CMT resulted in greater changes in the VAS score, that is, better pain relief. Table 4 summarizes the VAS scores for the patients in the 2 groups. Before the treatments, the VAS pain scores of the 2 groups did not differ significantly, that is, moderate pain was reported by 60% of patients in the CMT group and by 68% in the LTARM group. After 4 weeks of treatment, the VAS scores changed considerably in the CMT group, wherein 63% of patients felt no or mild pain, whereas the VAS scores for moderate to severe pain were even higher in the LTARM group for 81% of patients (*P* < 0.001). Table 5 summarizes the results of the factor analysis for SPADI. Before the treatments, there were no obvious differences in terms of the SPADI scores (pain, disability, and total SPADI) in both groups. However, after treatment for 4 weeks, there were significant differences in all 3 SPADI categories. The results for the 2 groups in terms of the pain, disability, and total SPADI scores were 0.33 ± 0.14 versus 0.45 ± 0.14 (*P* < 0.01), 0.35 ± 0.14 versus 0.44 ± 0.14 (*P* < 0.05), and 0.34 ± 0.14 versus 0.44 ± 0.14 (*P* < 0.01) for the CMT and LTARM groups, respectively. For both groups of patients, no treatment-associated adverse effects were reported during the whole course of the study.

**Table 2:**
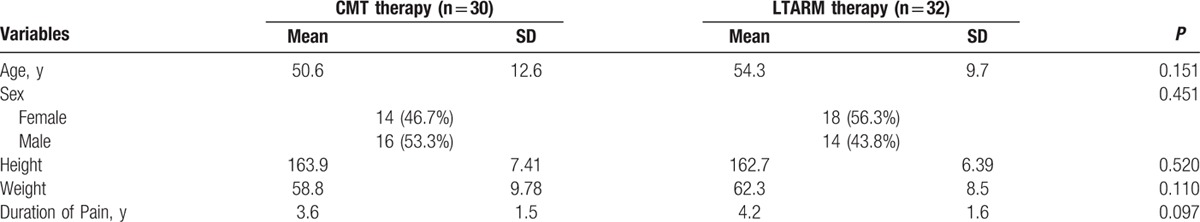
Baseline characteristics of patients.

**Table 3:**
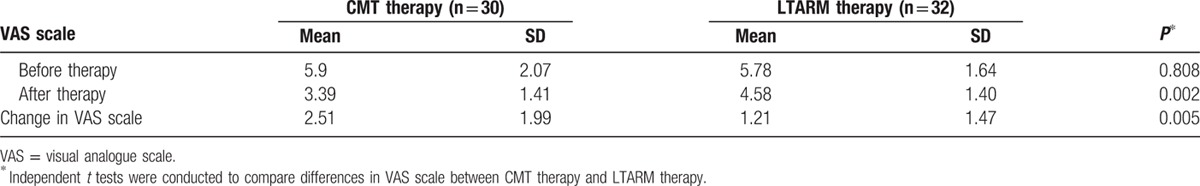
Overall changes in VAS scores after CMT and LTARM therapy.

**Table 4:**
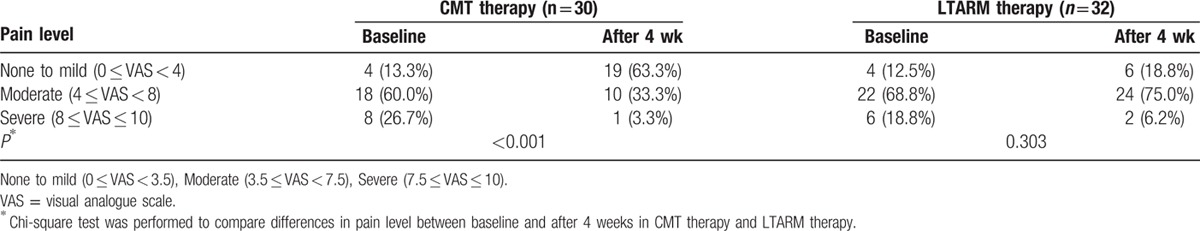
Comparison of the pain level distributions before and after treatment with CMT and LTARM.

**Table 5:**
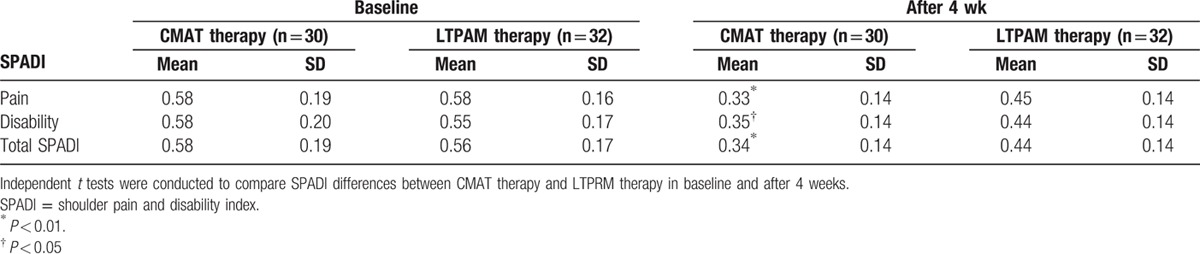
Results of factor analysis for SPADI.

## 4 Discussion

CMT provides an alternative for the treatment of different types of pain by using a systematic approach based on a variant of traditional Chinese acupuncture. CMT involves the manipulation of multiple sets of acupoints to relieve pain according to structured formulae using various techniques and tools, such as acupressure and EES.^[10]^ Many published CMT clinical reports have described the application of acupressure to treat various types of acute and chronic pains, such as dysmenorrhea and complex regional pain syndrome.^[16,17]^ However, to the best of our knowledge, only 1 previous study has described the application of EES for CMT, which focused on the effectiveness of EES in improving knee osteoarthritis pain and knee function.^[18]^ In our study, we demonstrated that by applying the technique with EES, CMT was more effective than LTARM for reducing shoulder pain of chronic myofascial origin.

Various noninvasive or minimally invasive techniques can be used for treating chronic shoulder pain, such as acupressure,^[19]^ dry needle,^[4]^ electroacupuncture,^[20]^ and transcutaneous electrical nerve stimulation (TENS).^[21,22]^ A common feature of these techniques is direct manipulation of the lesion site. All of these techniques have been used with varying degrees of success in the management of chronic shoulder pain. In a review, Chen and Wang^[19]^ showed that acupressure can reduce various types of pain, including dysmenorrhea, labor pain, lower back pain, chronic headache, and other traumatic pains. However, few if any previous studies have focused on the effectiveness of acupressure in treating shoulder pain. In particular, Chen and Wang^[19]^ aimed to establish a credible evidence base for the use of acupressure in treating various types of pain, but shoulder pain relief was conspicuously absent from their systematic review. However, numerous clinical case reports have shown that CMT is effective in pain management.^[10–12,16–18]^ For example, we successfully treated postlaparoscopic shoulder tip-pain with CMT by employing the acupressure technique.^[12]^ In the present study, we investigated the efficacy of CMT by applying the EES technique to treat chronic shoulder pain of myofascial origin, which we compared with the effects of LTARM.

In clinical practice, various techniques have been used to ensure the optimal stimulation of acupoints, such as acupuncture, electroacupuncture, and transcutaneous electric acupoint stimulation (TEAS).^[5,20–22]^ Compared with manual needling, electroacupuncture is a modern approach based on clinical and basic scientific evidence, which has advantages in terms of time savings and high reproducibility.^[23]^ Wang et al^[24]^ showed that acupoint stimulation by electroacupuncture or TEAS may have a similar therapeutic effect and underlying mechanism. In particular, it is considered that like electroacupuncture, TEAS can lead to a significant increase in analgesia by inducing the release of endogenous opioids.^[23,25]^ The tailor-made modality designed for CMT clinical application by our study group, which involves the application of EES to distal acupoints, can be considered as a modification of TEAS. Thus, it is possible that the underlying mechanism of CMT is at least partially similar to that of TEAS and electroacupuncture.

TENS is a form of noninvasive electrical stimulation that has been applied widely to treat various acute and chronic musculoskeletal pains via direct manipulation on painful areas. TENS evokes nonpainful electrical counter-stimulation via the afferent Aβ fibers and this “gate control” mechanism may interfere with nociception transmission to achieve an analgesic effect.^[26]^ Therefore, in our control group, EES could also be considered as a variant of TENS when applied directly to the affected areas. In our study, we compared local EES (in the LTARM group) and distal EES (in the CMT group), and both were shown to be analgesic, but the pain-relief effect was significantly better in the CMT group (VAS from 5.78 ± 1.64 to 4.58 ± 1.40; *P* < 0.005; SPADI pain subscale score from 0.58 ± 0.16 to 0.45 ± 0.14, *P* < 0.001) than that in the LTARM group. In terms of possible explanations, other mechanisms may be involved in addition to the “gate control” theory. Thus, the following hypothesis may be proposed. The mechanism known as diffuse noxious inhibitory control can modulate pain at a distance from the site of stimulation. It is possible that when producing peripheral stimulation, CMT might trigger a descending inhibition system that originates from the brainstem structures.^[27,28]^

According to the theory of traditional Chinese medicine, pain is a result of blood stasis due to Qi stagnation (a pathological alteration where a long-standing or severe obstruction of Qi impedes the blood flow, which is a situation characterized by the coexistence of Qi stagnation and blood stasis).^[29,30]^ In the CMT treatment, EES stimulates the distal healthy meridian to relieve shoulder pain. In other words, the stagnation of Qi is allowed to flow from the site of pain to the unobstructed or healthy meridian, thereby leading to the dissipation of shoulder pain. The C-point links the diseased meridian and treatment meridian, while the F-point is used for the treatment of the disease-associated symptoms or painful areas. By stimulating the C-point and F-point on a healthy meridian using acupressure, acupuncture, or EES, we can redirect the obstructed Qi from the diseased meridians in the painful regions to the aforementioned healthy meridian.

Zheng et al^[29]^ found that in patients who underwent multiple acupuncture sessions, the benefit of short-term pain relief and functional improvement might have been partly attributable to the so-called placebo effect. Similarly, the pain-alleviating effect attributed to EES in both groups may have been related to the specific and nonspecific effects of the therapeutic session, but we did not attempt to differentiate between these components in this study. Although it is debatable, some researchers have demonstrated that TENS has a placebo effect in reducing the overall pain intensity by about 20% to 30%.^[30,31]^ Moreover, this study specifically blinded the patients to their treatment. The reason for which they were blinded is to lower their bias in favor of the CMT treatment, thus eliminating the placebo effect in the overall study. Also, another more important reason is to distinguish which treatment is more effective between CMT and LTARM. However, due to methodological factors associated with the blinding of patients, it is difficult to distinguish the effects of TENS from the placebo effect. In our study, it was difficult to eliminate the possibility of placebo analgesia or to determine its level when applying EES to both groups.

### 4.1 Limitation

Our study had some limitations. First, all of the treatment procedures were prescribed by the same pain physician, so they were not double-blinded. However, we did exclude the physician from reviewing the outcomes, thereby single blinding the study. Second, the nonstandardized physical and daily activities of the patients might have affected the outcomes during the treatment period. Third, there was no sham group in our study, which made it difficult to address the possible issue of a placebo effect. It is known that sham therapy may be as efficacious as real therapy, or it may produce some degree of placebo effect, or no effect at all.^[32–34]^ In the worst case scenario, arranging a sham group would have put the patients under risk of unexpected adverse effects from long-term persistent pain. Finally, it would be desirable to conduct larger scale clinical trials to follow up the median or long-term effects of CMT in the future.

## 5 Conclusions

The current study demonstrated that CMT may be a more effective therapeutic option than LTARM for reducing chronic shoulder pain of myofascial origin in patients, where CMT achieved better functional recovery after 6 sessions over 4 weeks. Thus, on the basis of our results, CMT can be regarded as a special form of peripheral stimulation that achieves a neuromodulation effect. It may provide health care professionals with a noninvasive treatment modality to relieve chronic shoulder pain of myofascial origin, which can be utilized in addition to medications and physical therapy.
